# Mouse Embryonic Fibroblast Adipogenic Potential: A Comprehensive Transcriptome Analysis

**DOI:** 10.1080/21623945.2020.1859789

**Published:** 2020-12-21

**Authors:** Mohamed Al-Sayegh, Hamad Ali, Mohammad H Jamal, Mei ElGindi, Tina Chanyong, Khulood Al-Awadi, Mohamed Abu-Farha

**Affiliations:** a New York University Abu Dhabi, Division of Biology, Abu Dhabi, United Arab Emirates; b Department of Medical Laboratory Sciences, Faculty of Allied Health Sciences, Health Sciences Center (HSC), Kuwait University, Kuwait City, State of Kuwait; c Department of Genetics and Bioinformatics, Dasman Diabetes Institute (DDI), Kuwait City, State of Kuwait; d Department of Surgery, Faculty of Medicine, Health Sciences Center (HSC), Kuwait University, Kuwait City, State of Kuwait; e New York University Abu Dhabi, Design Studio, Abu Dhabi, United Arab Emirates; f Department of Biochemistry and Molecular Biology, Dasman Diabetes Institute (DDI), Kuwait City, State of Kuwait

**Keywords:** Adipose tissue, adipogenesis, adipocyte differentiation, mouse embryonic fibroblast, transcriptome

## Abstract

Our understanding of adipose tissue has progressed from an inert tissue for energy storage to be one of the largest endocrine organs regulating metabolic homoeostasis through its ability to synthesize and release various adipokines that regulate a myriad of pathways. The field of adipose tissue biology is growing due to this association with various chronic metabolic diseases. An important process in the regulation of adipose tissue biology is adipogenesis, which is the formation of new adipocytes. Investigating adipogenesis in vitro is currently a focus for identifying factors that might be utilized in clinically. A powerful tool for such work is high-throughput sequencing which can rapidly identify changes at gene expression level. Various cell models exist for studying adipogenesis and has been used in high-throughput studies, yet little is known about transcriptome profile that underlies adipogenesis in mouse embryonic fibroblasts. This study utilizes RNA-sequencing and computational analysis with DESeq2, gene ontology, protein–protein networks, and robust rank analysis to understand adipogenesis in mouse embryonic fibroblasts in-depth. Our analyses confirmed the requirement of mitotic clonal expansion prior to adipogenesis in this cell model and highlight the role of *Cebpa* and *Cebpb* in regulating adipogenesis through interactions of large numbers of genes.

## Introduction

Adipose Tissue (AT) function has advanced from its role as a primary storage of triglyceride in the form fat droplets for energy to the secretion of various signalling factors, known as adipokines that regulates various metabolic pathways. Thus, AT has been characterized as a major endocrine organ [1]. Adipogenesis, the transformation of preadipocytes into mature adipocytes, plays a critical role in development of specific functions and differentiation of cellular subtypes [2–4].

Adipocyte differentiation is a dynamic process tightly regulated through a multistep process conserved across species [2]. The process involves temporal expression of various signalling cascades that regulate the expression of transcription factors and thereby pro-adipogenic genes such as peroxisome proliferator-activated receptor-у (PPARG) [5,6]. Adipocyte differentiation is characterized by significant changes to cell morphology – from a fibroblast to a rounded spherical form – and acquisition of functional characteristics of AT cells [7]. These properties are directed by the expression of PPARG, which is central to adipogenesis. This receptor is encoded by *Pparg* and cooperate with members of the CCAAT/enhancer-binding proteins (CEBPs) family of transcription factors, specifically CEBP-β (CEBPB), encoded by *Cebpb*, and CEBP-α (CEBPA), encoded by *Cebpa*. Several studies have demonstrated the interplay among these factors during adipocyte differentiation and in enhancing adipogenic cell fate [8–11]. Mechanistically, induction of CEBPB, at an early phase of adipogenesis, promotes induction of PPARG and CEBPA by binding to their proximal promoter regions. Therefore, this results in activating several pro-adipogenic genes [12–16] that support the progression of preadipocytes cells into mature adipocytes [11,17].

Global gene profile changes in various murine cell models are reported in the literature that show the biological and clinical significance of adipogenesis through comprehensive analysis of differentially expressed genes (DEGs) using tools such as RNA sequencing (RNA-Seq) [18–24]. Through statistical significance and fold induction, the final lists of DEGs are further analysed using gene ontology (GO) analysis, and can assist in construction of protein–protein interactions (PPI) and gene networks. However, RNA-Seq data are variable, and biased analyses are possible. Thus, unbiased methods are necessary for generating transcriptome data to increase confidence of DEG lists. The robust rank aggregation (RRA) method that relies on algorithms that produce a significant score for genes that consistently rank better than expected has been applied to transcriptome data [25,26]. In this method estimates of significance are provided as *p*-values that assign probabilities showing that the differential expression of a given gene is related to adipogenesis [25,27]. *p*-values can be used to rank DEGs, where the lower the value, the higher its rank. An RRA approach allows multiple transcriptome profiles to be characterized by more reliable molecular targets.

Most knowledge of AT is based on in vitro studies of committed murine cell lines, notably 3T3-L1 and 3T3-F442A [28,29], and human cell lines, particularly mesenchymal stem cells (MSCs) [30,31]. However, some studies have demonstrated the adipogenic potential of mouse embryonic fibroblast (MEF) [16,32–34]. MEFs, isolated from 14-day-old mouse embryos (C57/BL6 52 strain), are classically used as ‘feeder layers’ for human and mouse embryonic stem cells, for DNA transfection assays, recombinant protein expression, and utilization for epigenome and transcriptome analysis (i.e. chromatin remodelling) [16,32]. Although MEFs can differentiate into multiple cell types, yet little is known in regard to specific mechanisms underlying their adipogenic potential.

In the current study, adipogenesis in MEFs transcriptome profiles was explored using generated DEGs at different timepoints. DEG lists relevant to adipose were generated based on statistical significance and used for an RRA method, and in construction of PPI and gene networks. Therefore, this study aims to utilizes transcriptome analysis methodology to acquire resourceful understanding in gene expression changes in MEFs that underlie adipocyte differentiation.

## Materials and methods

### Cell culture and in vitro adipocyte differentiation

MEFs cells were grown in 60-mm treated tissue culture dishes (Thermo Scientific). Cells were maintained in growth media (GM) containing Dulbecco’s Modified Eagle’s Medium (DMEM) (Gibco™) supplemented with 10% by volume of foetal bovine serum (FBS) (Corning) and 1% by volume of Pen/Strep/Glutamine (Gibco™) in a 5% CO_2_ atmosphere at 37°C. MEFs were seeded at 1 × 10^5^ cells per well in six-well tissue culture plates (Corning). At confluency, cells were induced into adipogenesis by incubating with differentiation media (DM) containing GM supplemented with 0.5 mM 3-isobutyl-1-methylxanthine (IBMX) (Sigma©), 10 μg/mL insulin (Sigma©), 1 μM dexamethasone (Sigma©) and 10 μM rosiglitazone (Sigma©). Cells were treated for 3 days (D3), followed by replacement of DM with GM, and incubation for another 2 days (D5).

### Oil red O staining and quantification

Differentiated and undifferentiated MEFs were washed with 1 ml of 1X PBS (Corning) once before fixing for 5 min with 4% paraformaldehyde in PBS at room temperature. After fixation, MEFs were washed three times with 1 ml of PBS and once with 60% isopropanol and were completely air-dried. MEFs were then stained with Oil Red O (ORO) [6 ORO:4 ddH_2_O ratio] (Sigma©) for 10 min at room temperature. Excess stain residue was removed with four ddH_2_O washes. Approximately 1 ml of PBS was then added for microscopic visualization. Images were processed at 460x magnification using a 20x objective lens, with transmitted bright-field light (EVOS® FLoid® Cell Imaging Station). For quantification, ORO stain particles were eluted with 100% isopropanol and analysed using Thermo Scientific™ Varioskan® Flash for spectrophotometry readings at 514 nm.

### RNA isolation and purification

Total RNA was extracted from MEFs using a combination of TriZol (Life Technologies™) and RNeasy Mini Kit (Qiagen©), with modifications from the manufacturer’s protocols. Cells in each well were washed once with 1 ml of PBS prior to the addition of 1 ml of TriZol reagent. TriZol lysates were added to fresh 1.5-ml tubes. Approximately 0.2 ml of chloroform was added per ml of TriZol and was centrifuged at a speed of 12,000 rpm at 4°C for 15 min. The upper aqueous phase (~400 ml in volume) was transferred to a fresh 1.5-ml tube. An equivalent volume of 70% ethanol was mixed with the RNA solution, and downstream purification was performed using a RNeasy Mini Spin Column (Qiagen©) according to the manufacturer’s protocol. RNA concentrations were determined using a Thermo Scientific™ NanoDrop 2000™.

### cDNA synthesis and quantitative PCR

Total isolated RNA (0.1 ng) was used as a template for synthesizing complementary DNA (cDNA) using a First-Strand Synthesis Kit (Invitrogen™) according to the manufacturer’s protocol. cDNA was diluted 1:50 in ddH_2_O prior to its use as a template for real-time PCR (RT-PCR) using SYBR Green (Thermo Scientific™). A total volume of 10 ul was prepared for the SYBR Green qPCR reaction [2.5 ul diluted cDNA product or nH_2_O, 2.5 ul 2 mM forward + reverse primer mix (Integrated DNA Technologies, Inc.) (Table 8) and 5 ul of SYBR Green]. The qPCR thermal cycle utilized an Applied Biosystems™ StepOnePlus™ platform, with one cycle of 50°C for 2 min and 95°C for 10 min followed by 44 cycles of 95°C for 15 s and 60°C for 1 min. Expression of gene targets values was normalized to housekeeping gene *β-actin* and was estimated using the ΔΔCt approach. Results represented on a log2 scale.

**Table 1.** GO terms under biological processes category of DEGs associated with fat or adipose development commonly identified between MEFD3plu/MEFD3min and MEFD5plu/MEFD5min

ID	GO Term	FDR (*p*-adj)	Gene Count
GO:0045444	fat cell differentiation	1.34E-10	108
GO:0050873	brown fat cell differentiation	6.21E-07	32
GO:0060612	adipose tissue development	0.003655776	18
GO:0045598	regulation of fat cell differentiation	0.00990133	41
GO:0045600	positive regulation of fat cell differentiation	0.0107074	22
GO:0045599	negative regulation of fat cell differentiation	0.016369719	10

**Table 2.** List of top 30 genes from the combined GO enriched terms related to fat or adipose development with the highest degrees

Gene	Degree	Betweenness Centrality	Closeness Centrality
*Akt1*	39	0.349267949	0.558282209
*Gsk3b*	23	0.230773486	0.478947368
*Adipoq*	21	0.101481567	0.484042553
*Cebpa*	21	0.072597239	0.484042553
*Ccnd1*	19	0.037552824	0.431279621
*Ppargc1a*	18	0.058255074	0.473958333
*Ucp1*	17	0.069045931	0.443902439
*Slc2a4*	17	0.041064493	0.473958333
*Fabp4*	16	0.063222749	0.443902439
*Retn*	15	0.073056209	0.443902439
*Vegfa*	15	0.041259118	0.446078431
*Cebpb*	14	0.041960465	0.455
*Lpl*	14	0.035363403	0.433333333
*Scd1*	14	0.022728141	0.433333333
*Xbp1*	11	0.027885025	0.397379913
*Sod2*	11	0.020179777	0.411764706
*Wnt10b*	11	0.01764364	0.411764706
*Dgat2*	10	0.003315263	0.380753138
*Sfrp1*	9	0.026403149	0.402654867
*Klf4*	9	0.015950399	0.40990991
*Snai2*	9	0.009428429	0.399122807
*Adrb3*	8	0.043638075	0.365461847
*Med1*	8	0.03520591	0.364
*Nr4a1*	7	0.032993776	0.393939394
*Hdac6*	7	0.008801478	0.399122807
*Sfrp2*	7	0.001072164	0.393939394
*Trib3*	6	0.012293217	0.400881057
*Gata2*	6	0.006169094	0.380753138
*Atf2*	6	0.003927372	0.40990991
*E2f1*	6	1.14E-04	0.397379913

**Table 3.** GO terms under biological processes category of the top 30 genes with the highest interaction degree

ID	GO Term	FDR	Gene Count
GO:0045444	fat cell differentiation	5.38E-49	28
GO:0045598	regulation of fat cell differentiation	1.23E-24	16
GO:0051240	positive regulation of multicellular organismal process	3.36E-18	25
GO:0009893	positive regulation of metabolic process	2.22E-16	28
GO:0010033	response to organic substance	4.49E-16	26

**Table 4.** List of DEGs observed gene interaction network of *Cebpa* at MEFD3plu/MEFD3min highlighting interaction type, expression fold change (*) and its significance (**), and significance of interaction (***)

Gene	Interactor Type	log2FC*	*p-*adj**	*p*-value***
*Trib1*	protein	1.2757261	9.53E-23	6.49E-24
*Trib2*	protein	0.2484696	0.04149	0.01775
*Sptan1*	protein	0.26594904	0.00596	0.002058
*Ppp1cb*	protein	−0.4120228	5.99E-04	1.71E-04
*Rfc2*	protein	−0.2496849	0.019862	0.007769
*Rfc3*	protein	−0.8750908	1.63E-12	1.98E-13
*Hdac1*	protein	−0.231246	0.022799	0.009052
*Rb1*	protein	1.30318645	1.01E-20	7.64E-22
*Nxf1*	protein	0.63601371	1.04E-09	1.56E-10
*Cebpa*	protein	4.81411526	1.00E-217	1.77E-220
*Smarca2*	protein	−1.3234689	3.98E-26	2.35E-27
*Ftsj3*	protein	0.69734207	3.12E-17	2.79E-18
*Baz1a*	protein	1.99860891	4.25E-116	2.82E-118
*Myh9*	protein	−0.7849686	1.03E-05	2.31E-06
*Rrp12*	protein	1.58576883	1.57E-31	7.57E-33
*Top1*	protein	0.46255199	1.14E-07	2.08E-08
*Nol10*	protein	1.13983894	3.93E-30	2.01E-31
*Myl6*	protein	−0.7482239	2.99E-06	6.31E-07
*Rbm39*	protein	0.18459507	0.004078	0.001357
*Safb2*	protein	0.60948244	0.004773	0.001614
*Mogs*	protein	0.30945873	0.003998	0.001328
*Hdgfrp2*	protein	0.62443954	1.88E-09	2.89E-10
*Thoc1*	protein	0.39876251	8.96E-04	2.63E-04
*Ddx54*	protein	0.39217318	0.009873	0.003584
*Rps14*	protein	−0.567406	0.003773	0.001246
*Dbt*	protein	1.59614098	3.93E-72	6.53E-74
*Rad21*	protein	0.75176143	4.90E-27	2.78E-28
*Vcp*	protein	0.29103786	1.91E-04	5.04E-05
*Snrnp70*	protein	0.23742331	0.013863	0.00521
*Rps4x*	protein	−0.4086177	0.03141	0.012959
*Pycr2*	protein	0.45103967	3.71E-05	8.92E-06
*Nup93*	protein	0.35399945	1.82E-05	4.23E-06
*Cdk1*	protein	−0.4306966	0.006843	0.002392
*Rrp1b*	protein	0.66632741	5.26E-07	1.02E-07
*Rps9*	protein	−0.321918	0.049547	0.021691
*Rpn1*	protein	−0.2017011	0.034541	0.014447
*Urb1*	protein	1.60300755	7.14E-21	5.33E-22
*Prpf4*	protein	0.48240813	2.17E-10	3.07E-11
*Gnb2*	protein	−0.4569276	2.17E-08	3.67E-09
*Cebpb*	protein	2.58063756	2.81E-74	4.55E-76
*Cdk4*	protein	−0.5755136	2.25E-09	3.49E-10
*Nop56*	protein	0.69728026	2.17E-17	1.93E-18
*Wdr5*	protein	0.3714022	0.001612	4.95E-04
*Ncoa3*	protein	−0.5646985	7.84E-04	2.27E-04
*Nat10*	protein	0.65085853	1.00E-07	1.82E-08
*Sap18*	protein	−0.2742982	0.011004	0.004035
*Pdcd11*	protein	0.8869514	1.21E-15	1.20E-16
*Rnps1*	protein	0.20642294	0.018317	0.007094
*D19Bwg1357e*	protein	0.4227897	1.77E-05	4.12E-06
*Ppp1ca*	protein	−0.5479237	2.22E-09	3.44E-10
*Psmd10*	protein	−0.4338942	0.005316	0.001815
*Asun*	protein	0.55150708	4.18E-10	6.09E-11
*Smarcd2*	protein	0.53243808	1.99E-09	3.06E-10
*Jdp2*	protein	−0.3267	2.78E-04	7.52E-05
*Zfr*	protein	−0.3860526	1.97E-08	3.32E-09
*Usp39*	protein	0.55563991	9.52E-08	1.72E-08
*Pnn*	protein	0.28073899	3.49E-05	8.37E-06
*Lig3*	protein	−0.3791107	4.94E-06	1.07E-06
*Stag1*	protein	−0.2677141	7.20E-06	1.59E-06
*Nol6*	protein	1.06249421	6.54E-10	9.71E-11
*Rpl27*	protein	0.52704131	7.16E-04	2.06E-04
*Lmnb1*	protein	−0.4357547	3.02E-07	5.74E-08
*Srsf5*	protein	0.54713539	5.07E-08	8.93E-09

**Table 5.** List of DEGs observed gene interaction network of *Cebpb* at MEFD3plu/MEFD3min highlighting interaction type, expression fold change (*) and its significance (**), and significance of interaction (***)

Gene	Interactor Type	log2FC*	*p*-adj**	*p*-value***
*Gsk3b*	protein	0.335455	0.032383	0.013407
*Rnf41*	protein	−0.54613	5.60E-10	8.27E-11
*Cebpa*	protein	4.814115	1.00E-217	1.77E-220
*Cebpb*	protein	2.580638	2.81E-74	4.55E-76
*Mapk14*	protein	0.916537	5.96E-16	5.79E-17
*Zbtb7b*	protein	0.591735	2.56E-10	3.65E-11
*Med1*	protein	0.428216	1.34E-04	3.47E-05
*Ptges2*	protein	1.653227	4.54E-71	7.80E-73
*Hdac1*	protein	−0.23125	0.022799	0.009052
*Rb1*	protein	1.303186	1.01E-20	7.64E-22
*Runx1t1*	protein	−0.9588	3.32E-10	4.78E-11

**Table 6.** List of DEGs observed gene interaction network of *Cebpa* at MEFD5plu/MEFD5min highlighting interaction type, expression fold change (*) and its significance (**), and significance of interaction (***)

Gene	Interactor Type	log2FC*	*p*-adj**	*p*-value***
*Trib3*	protein	0.62449	7.01E-09	7.70E-10
*Rfc3*	protein	−0.25539	0.048608	0.019505
*Ppp2r1a*	protein	−0.55163	7.48E-10	7.29E-11
*Cebpa*	protein	4.299336	4.97E-154	8.77E-157
*Srsf4*	protein	−0.71501	1.64E-04	3.54E-05
*Rbl2*	protein	−0.19147	0.025235	0.009225
*Baz1a*	protein	−0.5383	2.02E-09	2.05E-10
*Ncoa6*	protein	−0.51589	0.001752	4.70E-04
*Myh10*	protein	−1.32786	1.51E-29	3.04E-31
*Sin3a*	protein	−0.37961	7.85E-05	1.59E-05
*Top2a*	protein	−0.42784	1.12E-05	1.94E-06
*Lbr*	protein	−0.35716	3.73E-05	7.13E-06
*Noc2l*	protein	−0.85171	2.70E-20	9.75E-22
*Mogs*	protein	−0.35443	0.001064	2.72E-04
*Thoc2*	protein	0.384034	8.54E-06	1.45E-06
*Dbt*	protein	1.698034	8.17E-83	4.16E-85
*Vcp*	protein	−0.42803	2.42E-08	2.85E-09
*Sox9*	protein	−1.40303	2.12E-19	8.20E-21
*Cdk2*	protein	−0.52525	1.26E-11	1.00E-12
*Rrp1b*	protein	−1.08066	1.60E-16	7.82E-18
*Rfc5*	protein	−0.70548	4.01E-11	3.37E-12
*Rpn2*	protein	−0.39428	2.39E-06	3.72E-07
*Urb2*	protein	−0.79985	2.51E-09	2.59E-10
*Cebpb*	protein	0.906327	2.56E-09	2.65E-10
*Cdk5*	protein	0.307689	0.016229	0.005596
*Wdr6*	protein	−0.38057	0.020667	0.007347
*Ncoa4*	protein	1.158724	4.86E-14	3.00E-15
*Psmd11*	protein	−0.22202	4.70E-04	1.11E-04
*Zfr*	protein	0.340658	1.25E-06	1.87E-07
*Polr2a*	protein	−1.31882	2.31E-08	2.71E-09
*Mcm5*	protein	−1.02184	2.44E-25	6.26E-27
*Med24*	protein	−0.57037	1.56E-04	3.35E-05
*Stag2*	protein	0.404364	3.07E-04	7.00E-05
*Lmnb2*	protein	−0.81254	6.14E-14	3.83E-15

**Table 7.** List of DEGs observed gene interaction network of *Cebpb* at MEFD5plu/MEFD5min highlighting interaction type, expression fold change (*) and its significance (**), and significance of interaction (***)

Gene	Interactor Type	log2FC**	*p*-adj**	*p-*value***
*Gsk3b*	protein	−0.35866	0.023695	0.008603
*Sin3a*	protein	−0.37961	7.85E-05	1.59E-05
*Cebpa*	protein	4.299336	4.97E-154	8.77E-157
*Cebpb*	protein	0.906327	2.56E-09	2.65E-10
*Kmt2d*	protein	−0.98662	1.30E-04	2.76E-05
*Zbtb7b*	protein	0.343908	5.57E-04	1.34E-04
*Ptges3*	protein	−0.3568	3.20E-04	7.32E-05
*Bhlhe41*	protein	−1.80327	3.02E-11	2.51E-12

**Table 8.** List of primers

Gene	Primer	Sequence (5ʹ-3ʹ)
*Cebpa*	F	AAACAACGCAACGTGGAGA
	R	GCGGTCATTGTCACTGGTC
*Cebpb*	F	ATCGACTTCAGCCCCTACCT
	R	TAGTCGTCGGCGAAGAGG
*Fabp4 (aP2)*	F	GGATGGAAAGTCGACCACAA
	R	TGGAAGTCACGCCTTTCATA
*Lpl*	F	GGGAGTTTGGCTCCAGAGTTT
	R	TGTGTCTTCAGGGGTCCTTAG
*Adipoq*	F	TGTTCCTCTTAATCCTGCCCA
	R	CCAACCTGCACAAGTTCCCTT
*βactin*	F	CATTGCTGACAGGATGC
	R	TGCTGGAAGGTGGACA

### RNA-Seq library preparation and sequencing

Total RNA quality was estimated based on RNA integrity number (RIN) using Agilent BioAnalyzer 2100™. RNA samples with a RIN>8 was used for library preparation. RNA-Seq libraries were prepared using an Illumina® TruSeq Stranded mRNA Prep Kit accordingly with the manufacturer’s LS protocol. Samples were barcoded, multiplexed and sequenced (100 bp pair end) using the Illumina® NextSeq 550 platform at NYU Abu Dhabi (NYUAD) Genomic Core facility (Abu Dhabi, U.A.E).

### Transcriptome data generation

The DESeq2 computational pipeline was used to estimate raw count reads aligned to the reference genome [35]. Mouse genome (GRCm38/mm10) from the University of California Santa Cruz Genome Browser (https://genome.ucsc.edu/) was utilized as a reference [36]. Computing methods used a Linux-based command system on the New York University Abu Dhabi (NYUAD) high-performance computing (HPC) server platform Dalma (https://wikis.nyu.edu/display/ADRC/Cluster+-+Dalma). Raw data counts were deposited at National Centre for Biotechnology Information (NCBI) database and are available under Gene Expression Omnibus (GEO) accession number GSE152750.

### Bioinformatic and computational analysis

Heatmaps and correlation through PCA and distance heatmap dendrogram analysis were generated by RNA-Seq START (Shiny Transcriptome Analysis Resource Tool), via the New York University Abu Dhabi Centre of Genomic and Systems Biology (NYUAD-CGSB) Bioinformatics Online Analysis and Visualization Portal (http://tsar.abudhabi.nyu.edu/) [37].

DEG lists based on comparative analysis (i.e. statistical significance [*p*-value<0.05]) were identified using JMP Genomics software (http://www.jmp.com/software/genomics/). Selected gene lists were subjected to gene ontology (GO) term enrichment analysis using the g:Profiler database (https://biit.cs.ut.ee/gprofiler/gost) [38] or Database for Annotation, Visualization and Integrated Discover (DAVID) bioinformatics tool (https://david.ncifcrf.gov/home.jsp/) [39]. Enrichment terms are based on corrected *p*-adj or FDR of <0.05 and were therefore considered significant.

A PPI network of differentially expressed genes (DEGs) was constructed with the STRING database (http://www.string-db.org) followed by visualization with the Cytoscape software (version 3.6.0, Washington, DC, USA) (http://www.cytoscape.org/). The degree of nodes was determined with the plug-in Network Analysis app on the Cytoscape software. Gene interaction network models were constructed with the Biological General Repository for Interaction Datasets (BIOGRID; https://www.thebiogrid.org/) database followed by visualization of DEGs with the Cytoscape software.

### Statistical analysis

Statistical analysis used Student’s t-tests in Microsoft Excel™ (Microsoft®). Results are represented as the mean of at least three independent experiments, and a *p*-value<0.05 was considered significant.

## Result

### Formation of adipocyte-like cells from MEFs

**Figure 1.**
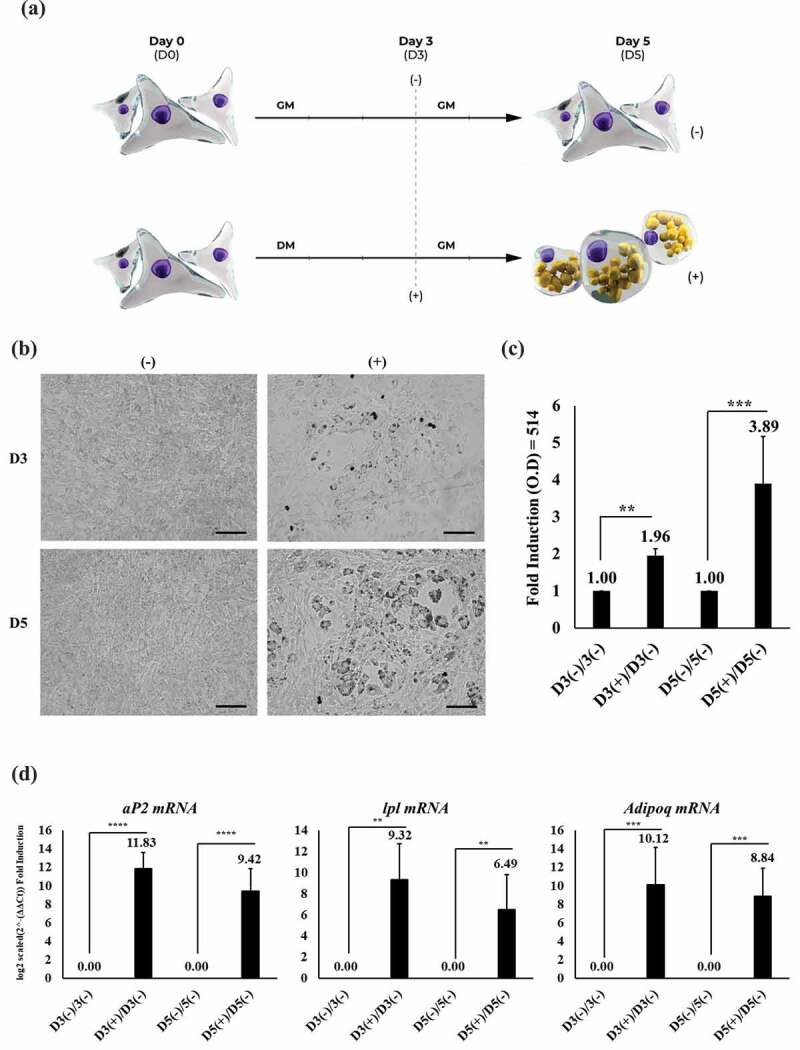
MEF capability to acquiring adipogenic features

We used MEFs in an initial incubation of cells – from day 0 (D0) – with adipogenic differentiating factors in culture medium (see Material and Method section) for 3 days (D3) followed by the removal of these factors and an additional incubation for 2 days (D5) (Figure 1a). Lipid accumulation was assessed with Oil Red O (ORO) staining on days 3 and 5. As expected, lipid accumulation was observed as droplet formation in treated (+) cells, but not in untreated (-) cells at both timepoints. Qualitatively, more and larger droplets were seen on D5 (Figure 1b). This finding was confirmed quantitatively using spectrophotometry with extracted stain. Higher optical density (OD) was recorded incrementally from treated cells, where OD was higher on D5 than on D3 (Figure 1c). We also examined endogenous expression of specific gene markers that are constitutively active in AT to confirm that results were not due to a random effect of treatment. Expression of mRNA levels for the adipocyte protein 2 (*aP2*) gene, also known as free fatty acid-binding protein 4 (*Fapb4*) [40] (Figure 1d; left chart), lipoprotein lipase (*Lpl*) gene [41] (Figure 1d; middle chart), and adiponectin (*Adipoq*) gene [42] (Figure 1d; right chart) were significantly upregulated after treatment. Comparatively, mRNA expression levels of the *aP2, Lpl*, and *Adipoq* genes were higher on D3, but not significantly, than on D5. These observations indicate that MEF developed adipogenic identity and functional activity. Thus, the process used to induce cells towards differentiation into adipocytes is a useful platform for transcriptome analysis.

(A) Schematic diagram illustrating experimental workflow of MEFs undergoing adipocyte differentiation in-vitro. Conditions include treated (+) condition with differentiation media (DM) and non-treated condition (-) in growth media (GM) from day 0 (D0) till day 3 (D3). Cells were further grown two more days (D5) following treatments. (B) ORO staining of represented timepoints; scales bar = 100 μm). (C) Quantitation of lipid accumulation based fold induction OD (514 nm) readings from ORO stains, illustrating MEFs at the binding timepoints D3(-)/D3(-) as well as D3(+)/D3(-) and later timepoints D5(-)/D5(-) as well as D5(+)D5(-) (n = 4; **P-Value<0.01, ***P-Value<0.001). (D) Quantitative RT-PCR analysis of *aP2* (left chart), *Lpl* (middle chart), and *Adipoq* gene during corresponding timepoints based on mRNA fold induction level and represented in log2 scaled form (n = 4; **P-Value<0.01, ***P-Value<0.001, ****P-Value<0.0001).

### Global differential gene expression changes

**Figure 2.**
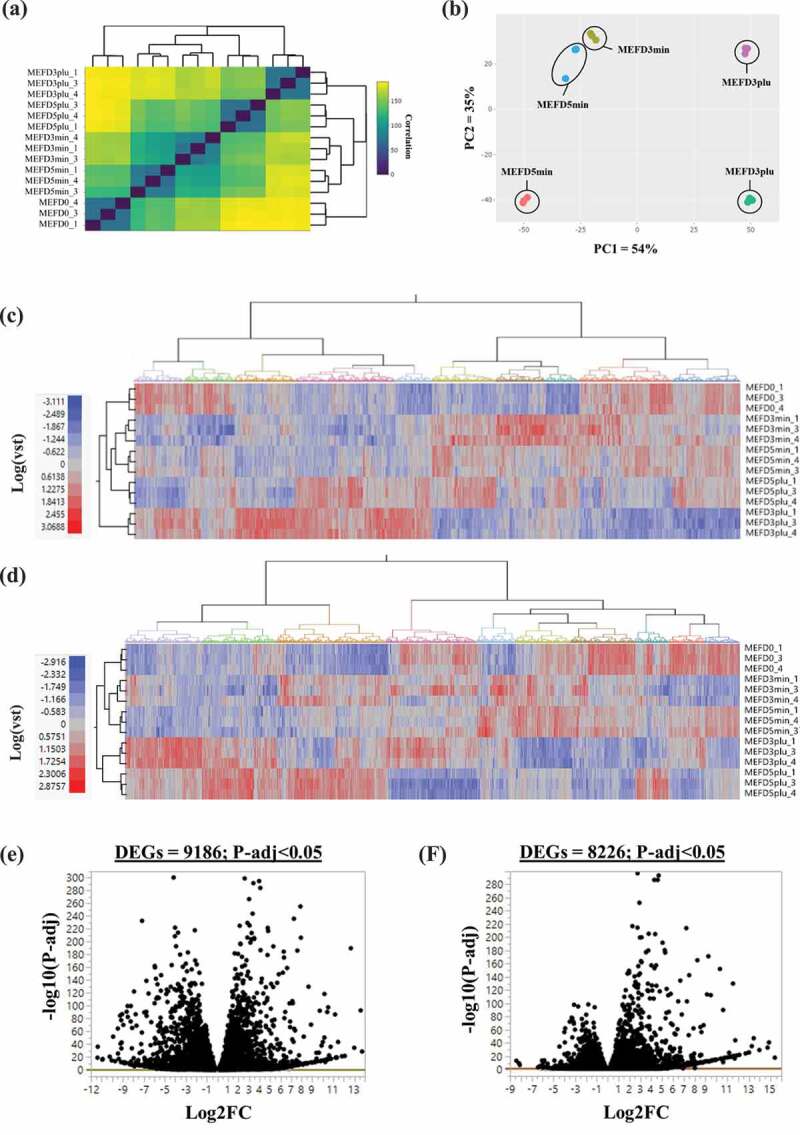
Correlation analysis and number of differentially expressed genes

Correlations were assessed with principle component analysis (PCA) and distance heatmap [43–45] for all timepoints to gain insight into changes at the transcriptome level. Times assessed included day 0 (MEFD0_1, MEFD0_3, and MEFD0_4), day 3 untreated (MEFD3min_1, MEFD3min_3, and MEFD3min_4), day 3 treated (MEFD3plu_1, MEFD3plu_3, and MEFD3plu_4), day 5 untreated (MEFD5min_1, MEFD5min_3, and MEFD5min_4), and day 5 treated (MEFD5plu_1, MEFD5plu_3, and MEFD5plu_4) (Figure 2a and B). Correlations between replicates for the same treatment condition showed tight clustering patterns (Figure 2a). According to PCA plot, these patterns where primarily driven by effect of treatments (PC1 = 58%) followed by time (PC2 = 31%) (Figure 2b).

Correlation analysis displayed in (A) distance heatmap, with legend representing Pearson correlation coefficient values between samples, and (B) principle component analysis based on data counts from RNA-Seqs prepared in triplicates for D0 (MEFD0), D3(-) (MEFD3min), D3(+) (MEFD3plu), D5(-) (MEFD5min) and D5(+) (MEFD5plu). Metric heatmap, based on log VST normalization value, displaying differential genes expression based on statistical significance of P-adj<0.05 for (C) MEFD3plu/MEFD3min (DEGs = 9186) and (D) MEFD5plu/MEFD5min (DEGs = 8226). Volcano plot for pair wise comparison for (E) MEFD3plu/MEFD3min and (F) MEFD5plu/MEFD5min. Number of DEGs expressions (normalized to Log2 fold change (Log2FC)) are displayed on each panel based on a P-adj cut-off of <0.05 (red line) with 9186 and 8226 genes for MEFD3plu/MEFD3min and MEFD5plu/MEFD5min.

Based on the correlation analysis, DEGs were identified for intermediate (MEFD3plu/MEFD3min) and terminal timepoints (MEFD5plu/MEFD5min). DEGs were genes that showed a statistically significant change in expression (*p*-adj<0.05) between treatments. Using volcano plots, a total of 9186 (Figure 2e) and 8226 (figure 2f) DEGs were identified for MEFD3plu/MEFD3min and MEFD5plu/MEFD5min, respectively. Differences in numbers of DEGs may reflect a series of synergetic processes, such as genes response to signalling molecules (i.e. cellular dynamics) or nuclear changes (i.e. methylation/acetylation events) during adipogenesis. Such processes may be involved in specifying cell fate or enhancing adipogenic features. DEGs from both timepoints were further analysed via log scaled variance-stabilizing transformation (VST) to generate a heatmap of hierarchal clustering. Expression of selected DEGs lists in each timepoint was analysed across all conditions (MEFD0, MEFD3min, MEFD3plu, MEFD5min, and MEFD5plu) and their corresponding replicates. Variable expression patterns were observed across conditions (Figure 2c and D), which may reflect potency of the differentiation treatment. However, between both timepoints, there were no significant changes in the expression patterns and number of DEGs list isolated from MEFD3plu/MEFD3min and MEFD5plu/MEFD5min.

### Gene ontology, expression, and network interaction analysis of genes related to fat differentiation and development

**Figure 3.**
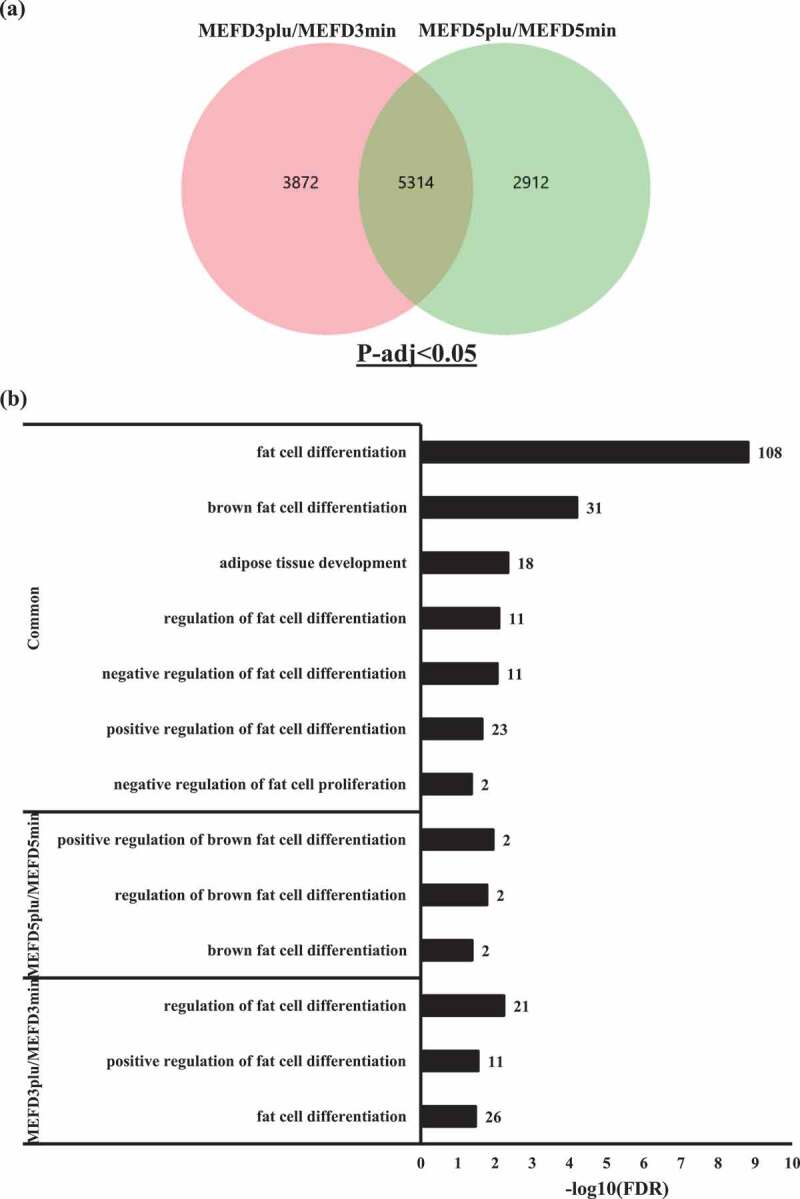
Differentially expressed genes (DEGs) GO term and PPI analysis

Functional aspects of DEGs were examined using gene GO databases. Firstly, DEGs from both timepoint sets were organized into a Venn diagram to eliminate redundancy of genes (Figure 3a). The diagram showed 5314 DEGs shared between MEFD3plu/MEFD3min and MEFD5plu/MEFD5min and 3872 and 2912 uniquely expressed genes between MEFD3plu/MEFD3min and MEFD5plu/MEFD5min, respectively.

(A) Venn diagram illustrating unique and common DEGs in MEFD3plu/MEFD3min and MEFD5plu/MEFD5min based on statistical significance cut-off p-adj<0.05. (B) GO term analysis (biological processes) of the common DEGs between MEFD3plu/MEFD3min and MEFD5plu/MEFD5min showing fat cell differentiation to have the highest enrichment significance. Number of genes displayed adjacently to in each enrichment bar chart. False dependent rate, represented as P-adj values, are normalized to -log10(P-adj).

The common 5314 DEGs showed 3224 enrichment terms overall. The enrichment list showed 92 enrichment terms related to differentiation processes, including neuron, chondrocyte, osteoblast, macrophage, myeloid, and various immune and progenitor cells. Interestingly, among enrichments, GO terms fat cell differentiation (GO:0045444, 108 genes) and brown fat cell differentiation (GO:0050873, 32 genes) were most significantly present. Further filtering of enrichment terms was performed with focused terms relevant to fat cells and adipose tissue (Table 1). Four additional GO terms related to fat cell and adipose development were identified (Figure 3b), including adipose tissue development (GO:0060612, 18 genes), regulation of fat cell differentiation (GO:0045598, 41 genes), positive regulation of fat cell differentiation (GO:0045600, 22 genes), and negative regulation of fat cell differentiation (GO:0045599, 10 genes) (top panel: Figure 3b; Table 1). MEFD3plu/MEF3min unique DEGs also relevant enrichments towards fat development including regulation of fat cell differentiation (GO:0045598,21 genes; Table S1), positive regulation of fat cell differentiation (GO:0045600, 11 genes; Table S2), and fat cell differentiation (GO:0045444, 26 genes; Table S3) (lower panel: Figure 3b). Whereas MEFD5plu/MEFD5min unique DEGs, constrictively showed enrichments towards brown fat cells including positive regulation of brown fat cell differentiation (GO:0090336, 2 genes; Table S4), regulation of brown fat cell differentiation (GO:0090335, 2 genes; Table S5), and brown fat cell differentiation (GO:0050873, 2 genes; Table S6) (middle panel: Figure 3b). Given that the common DEGs showed relevant enrichment terms as opposed to unique DEGs. Thus, the former was further analysed.

Expression patterns of the genes identified in the common enrichment list were further analysed using a metric heatmap (Supplementary Figure 2). The heatmap included all DEGs in each previous enrichment into a single list (119 genes), by removing redundant genes and normalizing gene counts on a log2 scale. The identified genes were categorized according to the timing of up or down differential expression (Supplementary Figure 2). Some genes were upregulated in both MEFD3plu and MEFD5plu, upregulated only in MEFD5plu, upregulated only in MEFD3plu, and downregulated in both MEFD5plu and MEFD3plu. The first category consisted of 15 genes, namely, *Rorc, Dgat2, Adipoq, Mrap, Fabp4, Lrg1, Lamb3, Cebpa, Adrb2, Retn, Zbtb16, Slc2a4, Ffar2, Fam57b*, and *Rgs2*. The second category consisted of 12 genes, namely, *Rarres2, Adrb3, Scd1, Plac8, Ucp1, Bnip3, Selenbp1, Wfdc21, Fabp3, Steap4, Psmb8*, and *Plac8*. The third category consisted of 13 genes, namely, *Ppargc1a, Cebpb, Arl4a, Pex11a, Aldh6a1, Sh2b2, Frzb, Fgf10, Nr4a3, Nr4a1, Cebpd, Smad3*, and *Sod2*. The fourth category consisted of 13 genes, namely, *Wnt10b, Msx2, Hmga2, Ccnd1, Htr2a, Wisp1, Inhbb, Tgfb1i1, Itga6, Metrnl, Alms1, E2f1*, and *Hdac6*.

**Figure 4.**
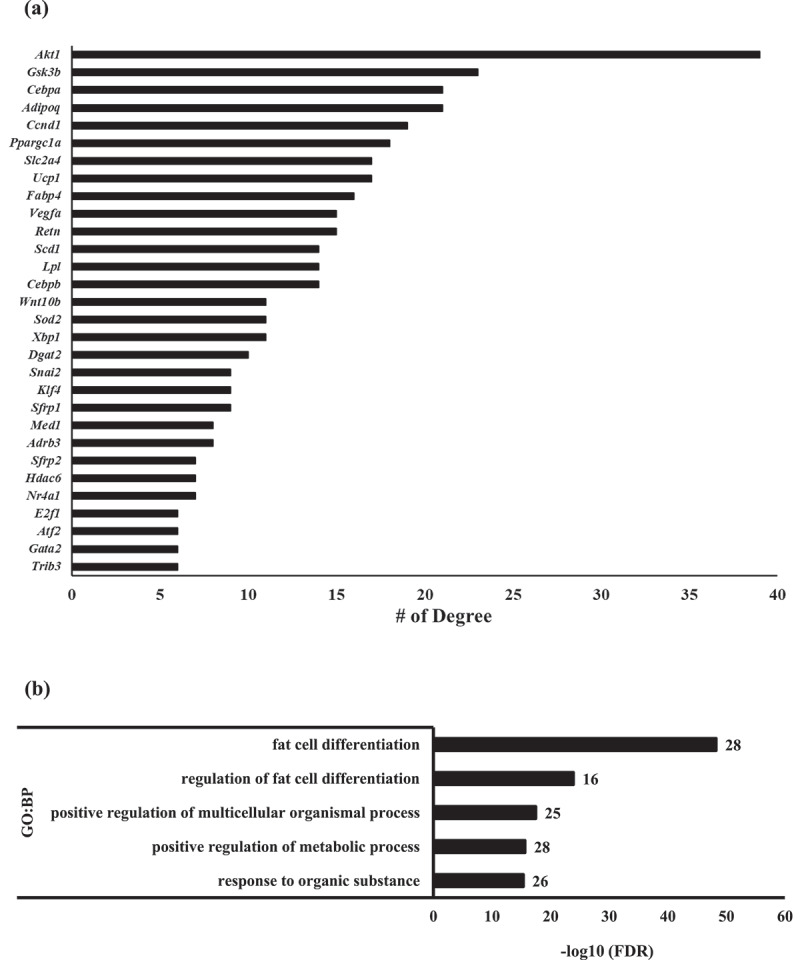
Degrees of centrality and GO term of DEGs

Specific interactions among these genes may be necessary for any given biological process. An interaction network is essential for understanding time-dependent regulation during differentiation observed in the previous heatmap with some genes. Thus, the relationship between gene expression patterns and timing is essential for elucidating fat differentiation and adipose development. Furthermore, key genes may be primary controllers of regulatory pathways. To explore this, a PPI network was constructed and curated via STRING and Cystoscape [46,47], respectively (Supplementary Figure 3). The numbers of degrees (adjacent links) from each node (gene) intersection were systematically selected with betweenness and closeness centrality (Figure 4a, Table 2 – displaying the top 30 genes). Gene *Akt1* showed the highest number of degrees, at 39, among all genes. *Adipoq* and *Fabp4* (*aP2*) showed 21 and 16 degrees, respectively, and *Ucp1* and *Lpl* showed 17 and 14 degrees, respectively. The top 30 genes from the GO term were further analysed at the biological process level with GO term analysis (Figure 4b) where most were associated with fat cell differentiation and metabolic and organic response processes (Table 3).

(A) Top right panel: List of the top 30 genes with the highest degrees in the PPI network. (B) GO term biological processes annotation of the top 30 genes with the highest degrees in the PPI network. Number of genes displayed adjacently to in each enrichment bar chart. False dependent rate, represented as P-adj values, are normalized to -log10(P-adj).

### *Gene interaction profile changes related to* Cebpa *and* Cebpb *expression*

**Figure 5.**
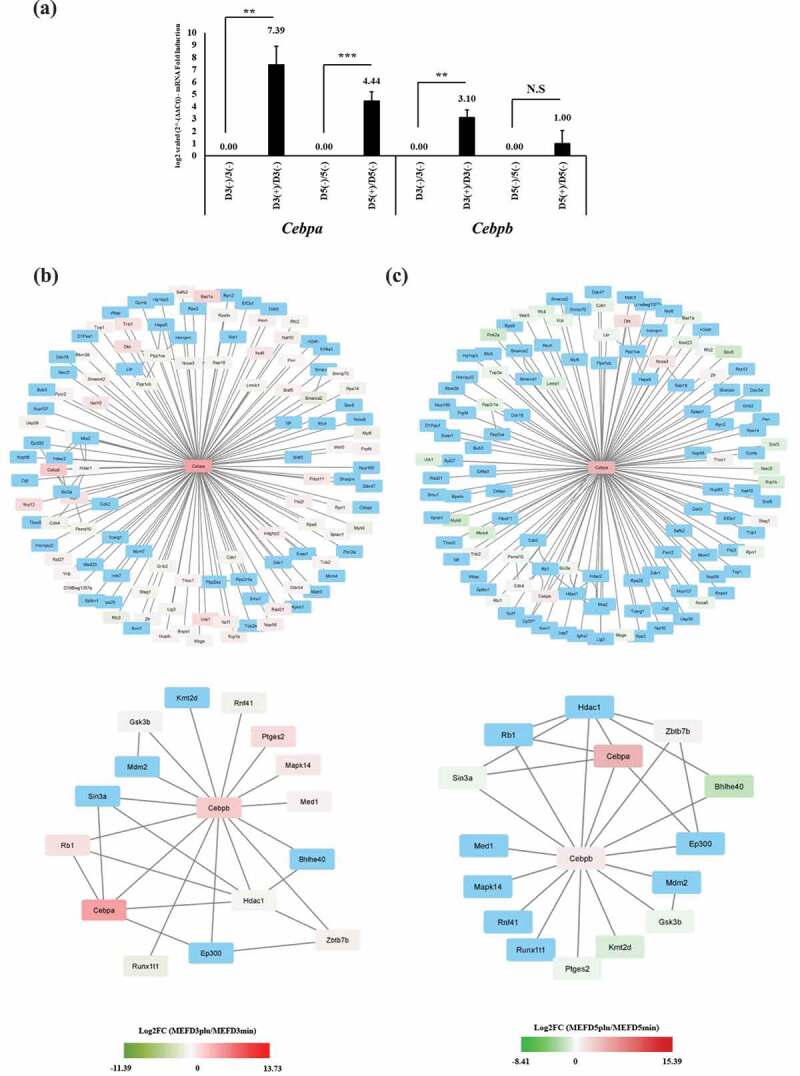
Verification of *Cebpa* and *Cebpb* gene expression and their association to other gene networks

Most genes identified in the PPI network are known to influence adipogenesis, and *Cebpa* and *Cebpb* were further investigated for their relation to protein factors. The endogenous expression of these factors may differ in terms of interaction with other proteins in a time-dependent manner (MEFD3plu/MEFD3min and MEFD5plu/MEFD5min). Therefore, *Cebpa* and *Cebpb* were examined with RT-PCR to determine and validate expression at the mRNA level (Figure 5a). On D3, significant upregulation of *Cebpa* and *Cebpb* was observed; however, on D5, only *Cebpa* showed significant upregulation (Figure 5a). These observations reflect the sensitivity of RNA-Seq analysis and suggest time-dependent interactions during treatment.

(A) Quantitative RT-PCR analysis of *Cebpa* and *Cebpb* gene during corresponding timepoints based on mRNA fold induction level and represented in log2 scaled form (n = 3; **P-Value<0.05; ***P-Value<0.01; N. S = not significant). (B) and (C) represent gene network associate’s models (from BioGRID) of *Cebpa* (upper panel in both B and C) and *Cebpb* (lower panel in both B and C) at MEFD3plu/MEFD3min and MEFD5plu/MEFD5min, respectively. Blue nodes are genes not represented in based on designated DEGs cut-off of P-adj<0.05; whereas colour nodes are genes identified based on cut-off. Varied colour intensities of nodes are based on log fold changes in each MEFD3plu/MEFD3min and MEFD5plu/MEFD5min (legend displayed under each panel condition).

Both *Cebpa* and *Cebpb* are noted for their pioneering effects in driving adipogenesis. Therefore, gene interaction networks were formulated for both genes and were comparatively analysed against DEGs list from each MEFD3plu/MEFD3min and MEFD5plu/MEFD5min lists. Gene interaction networks for murine sources were downloaded from BioGRID and uploaded to Cytoscape [47,48]. The *Cebpa* network, consisting of 117 interactions, 63 and 35 genes were observed in DEGs list of MEFD3plu/MEFD3min (upper panel: Figure 5b; Table 4) and MEFD5plu/MEFD5min (upper panel: Figure 5c; Table 6), respectively. Whereas in the *Cebpb* network, consisting of 16 interactions, 11 and 8 genes were observed in DEGs list of MEFD3plu/MEFD3min (lower panel: Figure 5b; Table 5) and MEFD5plu/MEFD5min (lower panel: Figure 4c; Table 7), respectively. Most interactions identified are directed through a protein targeted manner.

**Figure 6.**
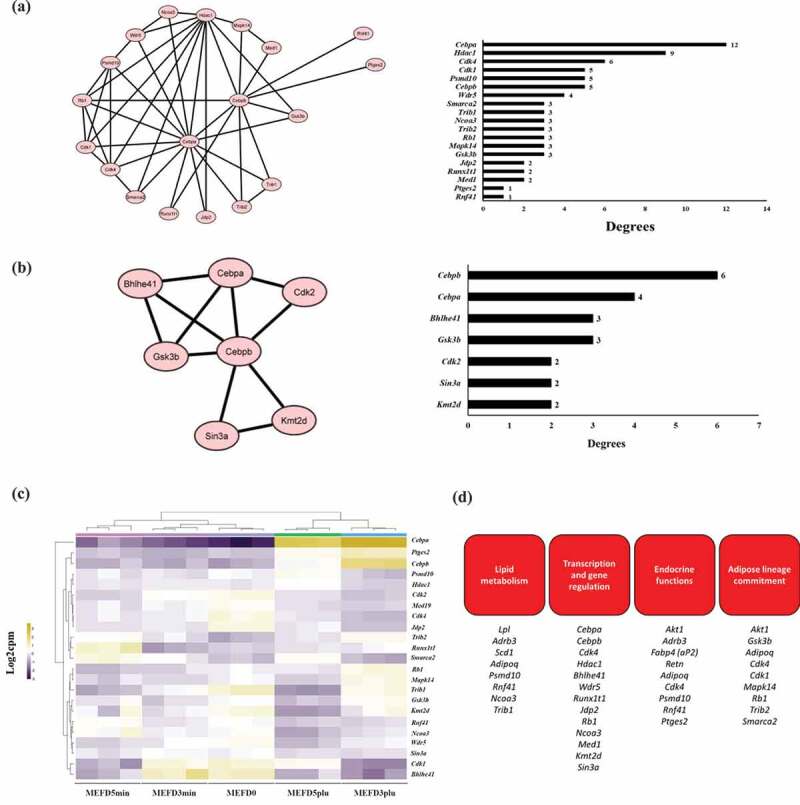
PPI network of *Cebpa* and *Cebpb* in relation to identified DEGs

To further decipher *Cebpa* and *Cebpb* interactions, PPI networks of the DEGs detected in each timepoint were constructed using STRING. Design of construct, formulated via Cytoscape, were restricted on direct interactions of DEG nodes with either *Cebpa* and *Cebpb* in each timepoint. The genes were ranked in degree of interaction in each timepoint after merging *Cebpa* and *Cebpb* profiles (left panels: Figure 6a and B). Based on the network maps, there were more direct interactions at day 3 (8 ranked orders) than that of day 5 (4 ranked orders). At day 3, *Cebpa* showed the highest level of interaction at 12 degree; whereas *Cebpb* was ranked in the fourth order, along with *Cdk1* and *Psmb10*, at 5 degrees (right panel: Figure 6a). Interestingly, *Hdac1* and *Cdk4* were ranked in the second and third order at 9 and 6 degrees, respectively. Moreover, *Wrd5* was ranked at the fifth order at 4 degrees; whereas *Smarca2, Trib1, Rb1, Mapk14*, and *Gsk3b* were collectively ranked in the sixth order with 3 degrees each. Further to those, *Jpr2, Runx1t1*, and *Med1* were also collectively ranked at seventh order at 2 degree; whereas *Pteg2* and *Rnf1* were ranked at the eighth order with a degree level of 1. Inversely, at day 3, *Cebpb* showed the highest level of interaction at 6 degrees followed by *Cebpa* at 4 degrees (right panel: Figure 6b). Genes *Bhlhe41* and *Gsk3b* were ranked at third order at 3 degrees each; whereas *Cdk2, Sin3a*, and *Kmt2d* were ranked at the fourth order with 2 degrees each. The expression of the genes was further analysed using a metric heatmaps, which showed variable patterns among the genes at different timepoints (MEFD0 – day 0, MEFD3min – untreated day 3, MEFD5min – untreated day 5, MEFD3plu – treated day 3, and MEFD5plu – treated day 5) (Figure 6c). As previously shown, *Cebpa, Cebpb*, and *Ptge2* were observed to be differentially upregulated in the treated conditions of day 3 and 5. Interesting, *Psmb10* and *Hdac1* were observed to be differentially upregulated in treated MEF at day 5 as opposed to that at day 3. Whereas, *Mapk14, Gsk3b, Trib1, Kmt2d*, and *Rb1* were differentially upregulated in treated MEFs at day 3 rather than day 5. There were also number of genes that were observed to be differentially downregulated due to the potency effect of differentiation treated, which included *Cdk2, Med19, Cdk4, Jdp2, Rnf41, Ncoa3, Wdr5, Sin3a, Cdk1*, and *Bhlhe41*. Overall, these variable observations between the different timepoints may suggest that specific interactions occur in a time-dependent manner for *Cebpa* and *Cebpb*.

(A) Left panel: a merged PPI network of *Cebpa* and *Cebpb* and direct associated nodes, identified as DEGs in MEFD3plu/MEFD3min. Chart highlighting level of interaction degrees of all nodes corresponding to adjacent PPI network map. (B) Left panel: a merged PPI network of *Cebpa* and *Cebpb* and direct associated nodes, identified as DEGs in MEFD5plu/MEFD5min. Right panel: Chart highlighting level of interaction degrees of all nodes corresponding to adjacent PPI network map. (C) Metric heatmap, based on VST normalization, displaying identified DEGs with direct interactions with *Cebpa* and *Cebpb* of all timepoints. (D) Biological features DEGs identified correspond to or play a functional role.

## Discussion

AT consists of various cell types, with adipocytes as the major cell type present in mature tissues. Progenitor populations of stem cells and preadipocytes are also present. These latter cells can differentiate into various cell types, including adipocytes and its corresponding subtypes. Proliferation and differentiation of preadipocyte to mature cells continually sustain and maintain AT function. AT is present throughout the life cycle, and 50% of human subcutaneous fat cells are renewed every 8 years [49], illustrating the necessity of dynamic adipocyte replenishment through adipogenesis. Furthermore, from a global public health perspective, dysfunction of AT is linked to chronic diseases/disorder (i.e. obesity, cardiovascular diseases, diabetes) [4]. Therefore, the study of adipogenesis in vitro at molecular and mechanistic levels is essential for development of better therapeutic targets. Many studies that elucidate the processes of adipogenesis have used different cell lines, such as 3T3-L1, 3T3-F442A, MSCs, murine and human adipose stem cells (ASCs), and MEFs [16,28–34]. Some of these same models have also been used to explore transcriptomic aspects of adipogenesis [16,19,20,50–52]. Though MEFs have been used extensively, yet little is known about the dynamics of adipogenesis at a global gene level.

MEFs are fibroblasts derived and isolated from mouse embryos. When cultured in vitro, MEFs display spindle-like features that are typical of many fibroblasts [53–55]. MEFs are used extensively as a cellular ‘scaffolding’ tool in stem cell biology in maintaining the growth of primitive undifferentiated cells such as mouse- and human-induced pluripotent or embryonic stem cells [55,56]. Moreover, in addition to adipocytes, MEFs display multilineage differentiation ability for the production of effector cell types, including osteocytes, chondrocytes and neurons [32–34,57–59]. Most studies of adipocytes illustrate MEF adipogenic potential through observation of cellular morphological changes, such as lipid accumulation during differentiation, or by evaluating endogenous expression of known gene markers. In this study, MEFs were also observed to develop morphological changes associated with adipogenesis. MEFs accumulated lipids, shown by ORO staining, known to target lipid triglyceride droplets [28,29,60]. This accumulation was measured quantitatively with spectrophotometry [60]. Temporal lipid accumulation was assessed at 0, 3, and 5 days in cells in non-treated, basal medium, and differentiation medium groups. These time points were chosen due to sufficiency of lipid formation in MEFs when exposed to differentiation factors, as observed previously [16]. Where continues incubation of MEFs with differentiation factors can lead continuous cell death [16], which will have biases towards exploring RNA-Seq data especially when targeting DEGs related to adipocyte or fat formation. Thus, a beginning (day 0), intermediate (day 3), and a terminal (day 5) time point was designated for this study. Further to this, the change from differentiation media to basal media was used to revive existing formed fat cells and explored its genotypic nature via transcriptome analysis. The differentiation media used in this study contained known inducing factors, including dexamethasone, insulin and 3-isobutyl-1-methylxanthine (Figure 1a). Although non-homogenous in lipid content, a positive linear increment in lipid droplets was seen in treated as opposed to non-treated cells. In parallel, similar increments were observed at the gene level with the expression of a lipolytic protein secreted during adipogenesis that is an in vivo feature of adipocytes including *aP2, Lpl*, and *Adipoq* [41,42,61]. Consistent with other studies, the present study observed formation of adipocyte-like features in vitro from MEFs under exposure of enhancing adipogenic factors. However, due to heterogenous population observed in MEFs, these observations do suggest that 3T3-L1 still remains to be an optimum model to study the functional aspect of adipogenesis and its metabolism. Yet, MEFs use as a model in AT development can be further explored and potentially utilized to study adipogenesis.

The ultimate aim of this study was to explore global gene expression controlled in vitro experiments. The study included analysis of transcriptome data based on statistical significance and ranking methods (RRA). Analysis of collected data offers valuable insight, biologically and clinically, to adipogenesis in a systematic fashion. Non-treated and treated MEFs showed distinct cell morphology, based on lipid accumulation, and expression of *aP2, Lpl*, and *Adipoq*. This finding is reflected in transcriptome data, where correlation analysis, according to distance heatmap and PCA plots, showed tight clustering between samples and corresponding replicates. The largest differences were found among treatment conditions and, to a lesser extent, with time. Thus, global changes do occur in the presence of inducers, as previously described.

Comparative pairwise analysis between treatments versus their undertreated counterpart (i.e. MEFD3plu/MEFD3min and MEFD5plu/MEFD5min) was used to identify DEGs. A *p*-adj cut-off value <0.05 was applied to eliminate nonsignificant fold expression and select genes in an unbiased fashion. The approach selects significant changes regardless of fold induction. A total of 9186 and 8226 DEGs were identified in MEFD3plu/MEFD3min and MEFD5plu/MEFD5min, respectively. These slight yet variable numbers between timepoints suggest that some genes are expressed at specific times during adipogenesis. Previous studies illustrate the formation of different subtypes of adipocytes (i.e. brown, white, and beige) during adipogenesis. White adipocytes are formed earlier than brown adipocytes [2,5,62,63]. Data from the present study will require further analysis to determine if the same is true for MEFs as they differentiate into adipocytes in vitro. Moreover, linking the latter to epigenetic regulation would be useful for complete understanding of MEF adipogenic potential [11,64,65]. However, this study focused on DEGs (5314) that commonly changed globally at different timepoints. GO term analysis of these DEGs showed significant enrichment for genes involved in fat cell differentiation and adipose tissue development. The GO terms associated with fat differentiation and AT development, 119 DEGs, was further analysed. The generated heatmap showed significant upregulation exclusively in treated cells for key genes known to be involved in adipogenesis [66–82], including *Mrap, Adig, Ffar2, Fabp4* (*aP2), Slc2a4, Lrg1, Zbtb16, Rorc, Lpl, Rarres2, Wfdc21, Adrb2, Adrb3, Steap4, Retn, Adipoq, Cebpa, Cebpb, Cebpd, Lamb3, Rgs2, Scd1* and *Fam57b*. Interesting, when filtering key terms associated towards differentiation, there was significant enrichment terms that were linked towards fat development. This can suggest the potent effectiveness of inducing and driving the expression of pro-adipogenic genes at the biological level. However, analysis of other categories, such cellular components and/or molecular function, would further shed light mechanistically of adipogenesis in MEFs.

The magnitude of expression of DEGs might affect their capacity to affect adipogenesis. However, other factors may also play central roles in various processes, such as maintaining growth, proliferation, and functionality of adipocytes during differentiation. Hence, a PPI network of DEGs based on RRA [26] identified through the GO terms was constructed, and the top 30 genes with the highest node degrees were selected. Several ranked genes are known to have major regulatory roles in adipogenesis. These genes include protein kinase B (PKB) gene (*Akt1*) (the highest-ranked gene), known as a major contributor for maintaining adipocyte and adipose tissue mass through insulin signalling [83]. Moreover, glycogen synthase kinase 3-β gene (*Gsk3b*) was identified. This gene negatively regulates the Wnt pathway during lineage commitment towards adipogenesis in mouse embryonic stem cells. The gene product inhibits retinoic acid receptor β and negatively inhibits brown adipocyte programming [84,85]. Interestingly, cyclin D1 (*Ccnd1*), a cell cycle regulator that downregulates during the early phases of adipogenesis in mesenchymal stem cells [86], is downregulated specifically in treated cells in the current study. Some DEGs, significantly induced with treatment, also showed high node degrees, including *Adipoq, Cebpa, Slc2a4, Fabp4 (aP2), Retn, Scd1, Lpl, Cebpb* and *Adrb3*. These genes are known as regulators of adipogenesis and are involved in lipid metabolism, transcription, and endocrine functions in adipocytes. Consistently, genes reported in this study reflect the magnitude of signalling necessary for adipogenesis and AT as functioning tissue. Results may support the use of MEFs as a cellular model for adipocyte differentiation in vitro. However, as mentioned previously, more in-depth study of GO terms in molecular function and cellular component categories, as well as KEGG pathways, might improve understanding of adipogenesis in MEFs.

Expression changes in key genes in the DEGs list of the GO term fat cell differentiation were further analysed for expression after treatment. *Cebpa* and *Cebpb* were selected based on their identification early in landscaping adipogenesis [3,9,13,87,88] and showed expression consistent with RNA-Seq data. These transcription factors play an essential role in promoting adipogenesis, along with the master regulator *Pparg* [9]. However, these genes can also be detrimental to the expression of subtype specificity [88]. Such effects may be due to differential expression in a time-dependent manner, as observed in this study. Specifically, some genes show variable interaction profiles with one CEBP factor depending on timepoint (day 3 vs day 5). For instance, 63 and 34 gene interactions for C*ebpa* were observed to be differentially expressed with MEFD3plu/MEFD3min and MEFD5plu/MEFD5min, respectively. Nine genes were observed to be common, and 54 and 25 genes were distributed uniquely with MEFD3plu/MEFD3min and MEFD5plu/MEFD5min conditions, respectively (Supplementary Figure 1a; Table 9). For *Cebpb*, 11 and 8 gene interactions were observed to be differentially expressed with MEFD3plu/MEFD3min and MEFD5plu/MEFD5min, respectively. Five genes were common, and six and three genes were distributed uniquely in MEFD3plu/MEFD3min and MEFD5plu/MEFD5min, respectively (Supplementary Figure 1b; Table 10).

**Table 9.** List of DEGs from Venn diagram (Supplementary Figure 1) highlighting common and unique genes under interactions with *Cebpa* (Table 9) and *Cebpb* (Table 10) in timepoints MEFD3plu/MEFD3min and MEFD5plu/MEFD5min

Conditions	DEGs
Common between MEFD3plu/MEF3min and MEFD5plu/MEFD5min	*Rfc3, Cebpa, Baz1a, Mogs, Dbt, Vcp, Rrp1b, Cebpb*, and *Zfr*
Unique in MEFD3plu/MEF3min	*Trib1, Trib2, Sptan1, Ppp1cb, Rfc2, Hdac1, Rb1, Nxf1, Smarca2, Ftsj3, Myh9, Rrp12, Top1, Nol10, Myl6, Rbm39, Safb2, Hdgfrp2, Thoc1, Ddx54, Rps14, Rad21, Snrnp70, Rps4x, Pycr2, Nup93, Cdk1, Rps9, Rpn1, Urb1, Prpf4, Gnb2, Cdk4, Nop56, Wdr5, Ncoa3, Nat10, Sap18, Pdcd11, Rnps1, D19Bwg1357e, Ppp1ca, Psmd10, Asun, Smarcd2, Jdp2, Usp39, Pnn, Lig3, Stag1, Nol6, Rpl27, Lmnb1* and *Srsf5*
Unique in MEFD5plu/MEFD5min	*Trib3, Ppp2r1a, Srsf4, Rbl2, Ncoa6, Myh10, Sin3a, Top2a, Lbr, Noc2l, Thoc2, Sox9, Cdk2, Rfc5, Rpn2, Urb2, Cdk5, Wdr6, Ncoa4, Psmd11, Polr2a, Mcm5, Med24, Stag2*, and *Lmnb2*
Common between MEFD3plu/MEF3min and MEFD5plu/MEFD5min	*Gsk3b, Cebpa, Cebpb, Zbtb7b* and *Ptges2*
Unique in MEFD3plu/MEF3min	*Rnf41, Mapk14, Med1, Hdac1, Rb1* and *Runx1t1*
Unique in MEFD5plu/MEFD5min	*Sin3a, Kmt2d* and *Bhlhe40*

The majority of genes and their interactions play pivotal roles in maintaining adipose tissue function and properties including lipid, glucose and caloric metabolism, regulation to adipocyte size and lineage commitment, epigenetic and gene transcription regulatory activities, and cell cycle regulation [86,89–105] (Supplementary Figure 4). For instance, the *Gsk3b* gene maintains adipogenic lineage commitment during differentiation of mouse embryonic fibroblasts [84] and shows the second-highest number of degrees in the PPI network. This gene was commonly observed in the current data at both timepoints, suggesting an important and conserved role in adipocyte differentiation. The marked sequential changes in the expression of several genes during differentiation treatment suggest initiation or inhibition of various pathways for many biological functions. In light of this, several studies have noted, and demonstrated in vitro, the reduced proliferation activity during adipogenesis [86,106–108]. For instance, the deletion of cyclin D1 (*Cdk1*) gene result in decrease expression of histone deacetylase (*Hdac1*) and increase of *Pparg* and therefore adipogenesis [109]. Although mitotic clonal expansion prior to adipogenesis remains to have conflicting views [110], these mechanisms have been characterized in murine models such as 3T3-L1 and mesenchymal stem cells [86,106]. In this study, similar trend of proliferation activity was observed in MEFs with *Hdac1* downregulated at day 3 of differentiation along with *Cdk1*. These downregulation effects were restored back at day 5, suggesting that the differentiation cocktail might play a potent inhibitory role. Interestingly, the PPI data in this study showed *Hdac1* to be highly ranked with 9 degrees at day 3, which also included direct interaction with *Cebpa* and *Cebpb*. Though the association of *Hdac1* and *Cebp* family of transcription factors have been observed in MEFs [111], 3T3-L1 [112], and other cell lines [113], this study systematically identified *Hdac1* having to play a pivotal role during adipogenesis in MEFs. Nonetheless, other factors identified here can attribute more to the nature aspect of adipogenesis in MEFs, which will require further analysis.

## Conclusion

This study highlights roles of *Cebpa* and *Cebpb* in regulating adipogenesis through interactions of large numbers of genes. Their expression was regulated in this study in a time-dependent manner. Nonetheless, characterizing relationship among interaction of identified genes with CEBP factors (or other pioneering factors) will be essential in future work. This study has demonstrated the strength in ranking method into stressing the most influential factors driving adipogenesis in MEFs via transcriptome data. Systematic global gene expression changes during adipogenesis allow us to better understand this process for future therapeutic targeting. In addition to studying known model for adipogenesis, such as 3T3-L1 and mesenchymal stem cells, this study illustrates the use of MEFs to better understand adipogenesis from transcriptome data by identifying new key proteins that can potentially regulate adipogenesis. As such, this study has systematically noted the main factor that drive differentiation was due to reduction in proliferation activity given the downregulation of cell cycle regulated genes and its associates. Indeed, further analysis from this study data will be needed to decipher MEFs adipogenic potential.

## References

[1] Negrel R, Grimaldi P, Ailhaud G. Establishment of preadipocyte clonal line from epididymal fat pad of ob/ob mouse that responds to insulin and to lipolytic hormones. Proc Natl Acad Sci U S A. 1978;75(12):6054–6058. 216011 10.1073/pnas.75.12.6054 PMC393116

[2] Rosen ED, MacDougald OA. Adipocyte differentiation from the inside out. Nat Rev Mol Cell Biol. 2006;7(12):885–896. 17139329 10.1038/nrm2066

[3] Gregoire FM, Smas CM, Sul HS. Understanding adipocyte differentiation. Physiol Rev. 1998;78(3):783–809. 9674695 10.1152/physrev.1998.78.3.783

[4] Bahmad HF, Daouk R, Azar J, et al. Modeling Adipogenesis: current and Future Perspective. Cells. 2020;9(10). doi:10.3390/cells9102326. PMC7590203 33092038

[5] Mota de Sa P, Richard AJ, Hang H, et al. Transcriptional Regulation of Adipogenesis. Compr Physiol. 2017;7(2):635–674. 28333384 10.1002/cphy.c160022

[6] Ntambi JM, Young-Cheul K. Adipocyte differentiation and gene expression. J Nutr. 2000;130(12):3122S–6S. 11110885 10.1093/jn/130.12.3122S

[7] Spiegelman BM, Flier JS. Adipogenesis and obesity: rounding out the big picture. Cell. 1996;87(3):377–389. 8898192 10.1016/s0092-8674(00)81359-8

[8] Morrison RF, Farmer SR. Insights into the transcriptional control of adipocyte differentiation. J Cell Biochem. 1999;75(Suppl 32–33):59–67. 10629104 10.1002/(sici)1097-4644(1999)75:32+<59::aid-jcb8>3.3.co;2-t

[9] Farmer SR. Regulation of PPARgamma activity during adipogenesis. Int J Obes (Lond). 2005;29(Suppl 1):S13–6. 15711576 10.1038/sj.ijo.0802907

[10] Farmer SR. Transcriptional control of adipocyte formation. Cell Metab. 2006;4(4):263–273. 17011499 10.1016/j.cmet.2006.07.001 PMC1958996

[11] Guo L, Li X, Tang QQ. Transcriptional regulation of adipocyte differentiation: a central role for CCAAT/enhancer-binding protein (C/EBP) beta. J Biol Chem. 2015;290(2):755–761. 25451943 10.1074/jbc.R114.619957 PMC4294498

[12] Wu Z, Rosen ED, Brun R, et al. Cross-regulation of C/EBP alpha and PPAR gamma controls the transcriptional pathway of adipogenesis and insulin sensitivity. Mol Cell. 1999;3(2):151–158. 10078198 10.1016/s1097-2765(00)80306-8

[13] Rosen ED, Walkey CJ, Puigserver P, et al. Transcriptional regulation of adipogenesis. Genes Dev. 2000;14(11):1293–1307. 10837022

[14] Rosen ED, Hsu CH, Wang X, et al. C/EBPalpha induces adipogenesis through PPARgamma: a unified pathway. Genes Dev. 2002;16(1):22–26. . 11782441 10.1101/gad.948702 PMC155311

[15] Tanaka T, Yoshida N, Kishimoto T, et al. Defective adipocyte differentiation in mice lacking the C/EBPbeta and/or C/EBPdelta gene. Embo J. 1997;16(24):7432–7443. 9405372 10.1093/emboj/16.24.7432 PMC1170343

[16] Al-Sayegh MA, Mahmood SR, Abul Khair SB, et al. beta-actin contributes to an open chromatin for activation of the adipogenic pioneer factor CEBPA during transcriptional reprograming. Mol Biol Cell. 2020;31(23):mbcE19110628. 10.1091/mbc.E19-11-0628 PMC7851876 32877276

[17] Tang QQ, Lane MD. Activation and centromeric localization of CCAAT/enhancer-binding proteins during the mitotic clonal expansion of adipocyte differentiation. Genes Dev. 1999;13(17):2231–2241. 10485846 10.1101/gad.13.17.2231 PMC316997

[18] Prokesch A, Hackl H, Hakim-Weber R, et al. Novel insights into adipogenesis from omics data. Curr Med Chem. 2009;16(23):2952–2964. 19689276 10.2174/092986709788803132 PMC2765082

[19] Xin Y, Li C, Guo Y, et al. RNA-Seq analysis reveals a negative role of MSMO1 with a synergized NSDHL expression during adipogenesis of 3T3-L1. Biosci Biotechnol Biochem. 2019;83(4):641–652. 30582412 10.1080/09168451.2018.1559719

[20] Ahmed M, Kim DR. Modelling the gene expression and the DNA-binding in the 3T3-L1 differentiating adipocytes. Adipocyte. 2019;8(1):401–411. 31809632 10.1080/21623945.2019.1697563 PMC6948977

[21] Basu U, Romao JM, Guan le L. Adipogenic transcriptome profiling using high throughput technologies. J Genomics. 2013;1:22–28. 25031652 10.7150/jgen.3781 PMC4091434

[22] Mikkelsen TS, Xu Z, Zhang X, et al. Comparative epigenomic analysis of murine and human adipogenesis. Cell. 2010;143(1):156–169. . 20887899 10.1016/j.cell.2010.09.006 PMC2950833

[23] Lee JE, Schmidt H, Lai B, et al. Transcriptional and epigenomic regulation of adipogenesis. Mol Cell Biol. 2019;39(11). doi:10.1128/MCB.00601-18 PMC6517598 30936246

[24] Shaik S, Martin EC, Hayes DJ, et al. Transcriptomic profiling of adipose derived stem cells undergoing osteogenesis by RNA-Seq. Sci Rep. 2019;9(1):11800. 31409848 10.1038/s41598-019-48089-1 PMC6692320

[25] Kolde R, Laur S, Adler P, et al. Robust rank aggregation for gene list integration and meta-analysis. Bioinformatics. 2012;28(4):573–580. 22247279 10.1093/bioinformatics/btr709 PMC3278763

[26] Zhang S, Wang L, Li S, et al. Identification of potential key genes associated with adipogenesis through integrated analysis of five mouse transcriptome datasets. Int J Mol Sci. 2018;19(11).doi:10.3390/ijms19113557. PMC6274731 30424473

[27] Li W, Xu H, Xiao T, et al. MAGeCK enables robust identification of essential genes from genome-scale CRISPR/Cas9 knockout screens. Genome Biol. 2014;15(12):554. . 25476604 10.1186/s13059-014-0554-4 PMC4290824

[28] Green H, Kehinde O. An established preadipose cell line and its differentiation in culture. II. Factors affecting the adipose conversion. Cell. 1975;5(1):19–27. 165899 10.1016/0092-8674(75)90087-2

[29] Green H, Kehinde O. Spontaneous heritable changes leading to increased adipose conversion in 3T3 cells. Cell. 1976;7(1):105–113. 949738 10.1016/0092-8674(76)90260-9

[30] Fink T, Zachar V. Adipogenic differentiation of human mesenchymal stem cells. Methods Mol Biol. 2011;698:243–251. 21431524 10.1007/978-1-60761-999-4_19

[31] Munir H, Ward LSC, Sheriff L, et al. Adipogenic differentiation of mesenchymal stem cells alters their immunomodulatory properties in a tissue-specific manner. Stem Cells. 2017;35(6):1636–1646. . 28376564 10.1002/stem.2622 PMC6052434

[32] Dastagir K, Reimers K, Lazaridis A, et al. Murine embryonic fibroblast cell lines differentiate into three mesenchymal lineages to different extents: new models to investigate differentiation processes. Cell Reprogram. 2014;16(4):241–252. 25068630 10.1089/cell.2014.0005 PMC4115680

[33] Fei Z, Bera TK, Liu X, et al. Ankrd26 gene disruption enhances adipogenesis of mouse embryonic fibroblasts. J Biol Chem. 2011;286(31):27761–27768. 21669876 10.1074/jbc.M111.248435 PMC3149366

[34] Baudry A, Yang ZZ, Hemmings BA. PKBalpha is required for adipose differentiation of mouse embryonic fibroblasts. J Cell Sci. 2006;119(Pt 5):889–897. 16478789 10.1242/jcs.02792

[35] Love MI, Huber W, Anders S. Moderated estimation of fold change and dispersion for RNA-seq data with DESeq2. Genome Biol. 2014;15(12):550. 25516281 10.1186/s13059-014-0550-8 PMC4302049

[36] Lee CM, Barber GP, Casper J, et al. UCSC Genome Browser enters 20th year. Nucleic Acids Res. 2019. doi:10.1093/nar/gkz1012. PMC7145642 31691824

[37] Nelson JW, Sklenar J, Barnes AP, et al. The START App: a web-based RNAseq analysis and visualization resource. Bioinformatics. 2017;33(3):447–449. 28171615 10.1093/bioinformatics/btw624 PMC6075080

[38] Raudvere U, Kolberg L, Kuzmin I, et al. g:Profiler: a web server for functional enrichment analysis and conversions of gene lists (2019 update). Nucleic Acids Res. 2019;47(W1):W191–W8. . 31066453 10.1093/nar/gkz369 PMC6602461

[39] Huang da W, Sherman BT, Lempicki RA. Bioinformatics enrichment tools: paths toward the comprehensive functional analysis of large gene lists. Nucleic Acids Res. 2009;37(1):1–13. 19033363 10.1093/nar/gkn923 PMC2615629

[40] Garin-Shkolnik T, Rudich A, Hotamisligil GS, et al. FABP4 attenuates PPARgamma and adipogenesis and is inversely correlated with PPARgamma in adipose tissues. Diabetes. 2014;63(3):900–911. 24319114 10.2337/db13-0436

[41] Wong H, Schotz MC. The lipase gene family. J Lipid Res. 2002;43(7):993–999. 12091482 10.1194/jlr.r200007-jlr200

[42] Oh DK, Ciaraldi T, Henry RR. Adiponectin in health and disease. Diabetes Obes Metab. 2007;9(3):282–289. 17391153 10.1111/j.1463-1326.2006.00610.x

[43] Son K, Yu S, Shin W, et al. A simple guideline to assess the characteristics of RNA-seq data. Biomed Res Int. 2018;2018:2906292. 30519573 10.1155/2018/2906292 PMC6241233

[44] Koch CM, Chiu SF, Akbarpour M, et al. A beginner’s guide to analysis of RNA sequencing data. Am J Respir Cell Mol Biol. 2018;59(2):145–157. . 29624415 10.1165/rcmb.2017-0430TR PMC6096346

[45] Chatterjee A, Ahn A, Rodger EJ, et al. A guide for designing and analyzing RNA-Seq data. Methods Mol Biol. 2018;1783:35–80. 29767357 10.1007/978-1-4939-7834-2_3

[46] Szklarczyk D, Gable AL, Lyon D, et al. STRING v11: protein-protein association networks with increased coverage, supporting functional discovery in genome-wide experimental datasets. Nucleic Acids Res. 2019;47(D1):D607–D13. . 30476243 10.1093/nar/gky1131 PMC6323986

[47] Shannon P, Markiel A, Ozier O, et al. Cytoscape: a software environment for integrated models of biomolecular interaction networks. Genome Res. 2003;13(11):2498–2504. . 14597658 10.1101/gr.1239303 PMC403769

[48] Oughtred R, Stark C, Breitkreutz BJ, et al. The BioGRID interaction database: 2019 update. Nucleic Acids Res. 2019;47(D1):D529–D41. . 30476227 10.1093/nar/gky1079 PMC6324058

[49] Spalding KL, Arner E, Westermark PO, et al. Dynamics of fat cell turnover in humans. Nature. 2008;453(7196):783–787. . 18454136 10.1038/nature06902

[50] Huo JS, McEachin RC, Cui TX, et al. Profiles of growth hormone (GH)-regulated genes reveal time-dependent responses and identify a mechanism for regulation of activating transcription factor 3 by GH. J Biol Chem. 2006;281(7):4132–4141. . 16326703 10.1074/jbc.M508492200

[51] Cho KA, Park M, Kim YH, et al. RNA sequencing reveals a transcriptomic portrait of human mesenchymal stem cells from bone marrow, adipose tissue, and palatine tonsils. Sci Rep. 2017;7(1):17114. 29214990 10.1038/s41598-017-16788-2 PMC5719355

[52] Jaager K, Islam S, Zajac P, et al. RNA-seq analysis reveals different dynamics of differentiation of human dermis- and adipose-derived stromal stem cells. PLoS One. 2012;7(6):e38833. 22723894 10.1371/journal.pone.0038833 PMC3378616

[53] Singhal PK, Sassi S, Lan L, et al. Mouse embryonic fibroblasts exhibit extensive developmental and phenotypic diversity. Proc Natl Acad Sci U S A. 2016;113(1):122–127. 26699463 10.1073/pnas.1522401112 PMC4711836

[54] Amand MMS, Hanover JA, Shiloach J. A comparison of strategies for immortalizing mouse embryonic fibroblasts. J Biol Methods. 2016;3(2):e41. 31453208 10.14440/jbm.2016.110 PMC6706133

[55] Xu J. Preparation, culture, and immortalization of mouse embryonic fibroblasts. Curr Protoc Mol Biol. 2005; 70(1):1. Chapter 28:Unit 28. 10.1002/0471142727.mb2801s70 18265366

[56] Takahashi K, Yamanaka S. Induction of pluripotent stem cells from mouse embryonic and adult fibroblast cultures by defined factors. Cell. 2006;126(4):663–676. 16904174 10.1016/j.cell.2006.07.024

[57] Oh SI, Park HS, Hwang I, et al. Efficient reprogramming of mouse fibroblasts to neuronal cells including dopaminergic neurons. ScientificWorldJournal. 2014;2014:957548. 24991651 10.1155/2014/957548 PMC4058809

[58] Ruiz-Ojeda FJ, Ruperez AI, Gomez-Llorente C, et al. Cell models and their application for studying adipogenic differentiation in relation to obesity: a review. Int J Mol Sci. 2016;17(7). doi:10.3390/ijms17071040 PMC4964416 27376273

[59] Hakim-Weber R, Krogsdam AM, Jorgensen C, et al. Transcriptional regulatory program in wild-type and retinoblastoma gene-deficient mouse embryonic fibroblasts during adipocyte differentiation. BMC Res Notes. 2011;4:157. 21615920 10.1186/1756-0500-4-157 PMC3127957

[60] Kraus NA, Ehebauer F, Zapp B, et al. Quantitative assessment of adipocyte differentiation in cell culture. Adipocyte. 2016;5(4):351–358. 27994948 10.1080/21623945.2016.1240137 PMC5160397

[61] Ertunc ME, Sikkeland J, Fenaroli F, et al. Secretion of fatty acid binding protein aP2 from adipocytes through a nonclassical pathway in response to adipocyte lipase activity. J Lipid Res. 2015;56(2):423–434. 25535287 10.1194/jlr.M055798 PMC4306695

[62] Lowe CE, O’Rahilly S, Rochford JJ. Adipogenesis at a glance. J Cell Sci. 2011;124(Pt 16):2681–2686. 21807935 10.1242/jcs.079699

[63] Stout MB, Swindell WR, Zhi X, et al. Transcriptome profiling reveals divergent expression shifts in brown and white adipose tissue from long-lived GHRKO mice. Oncotarget. 2015;6(29):26702–26715. . 26436954 10.18632/oncotarget.5760 PMC4694946

[64] Li HX, Xiao L, Wang C, et al. Review: epigenetic regulation of adipocyte differentiation and adipogenesis. J Zhejiang Univ Sci B. 2010;11(10):784–791. 20872986 10.1631/jzus.B0900401 PMC2950241

[65] Siersbaek R, Nielsen R, Mandrup S. Transcriptional networks and chromatin remodeling controlling adipogenesis. Trends Endocrinol Metab. 2012;23(2):56–64. 22079269 10.1016/j.tem.2011.10.001

[66] Kim NS, Kim YJ, Cho SY, et al. Transcriptional activation of melanocortin 2 receptor accessory protein by PPARgamma in adipocytes. Biochem Biophys Res Commun. 2013;439(3):401–406. 23994134 10.1016/j.bbrc.2013.08.061

[67] Hong YH, Hishikawa D, Miyahara H, et al. Up-regulation of adipogenin, an adipocyte plasma transmembrane protein, during adipogenesis. Mol Cell Biochem. 2005;276(1–2):133–141. 16132694 10.1007/s11010-005-3673-0

[68] Ivan J, Major E, Sipos A, et al. The short-chain fatty acid propionate inhibits adipogenic differentiation of human chorion-derived mesenchymal stem cells through the free fatty acid receptor 2. Stem Cells Dev. 2017;26(23):1724–1733. . 28992793 10.1089/scd.2017.0035 PMC5706617

[69] Fatima LA, Campello RS, Barreto-Andrade JN, et al. Estradiol stimulates adipogenesis and Slc2a4/GLUT4 expression via ESR1-mediated activation of CEBPA. Mol Cell Endocrinol. 2019;498:110447. 31100494 10.1016/j.mce.2019.05.006

[70] Haku S, Wakui H, Azushima K, et al. Early Enhanced Leucine-Rich alpha-2-Glycoprotein-1 Expression in Glomerular Endothelial Cells of Type 2 Diabetic Nephropathy Model Mice. Biomed Res Int. 2018;2018:2817045. 30515388 10.1155/2018/2817045 PMC6236974

[71] Wei S, Zhang M, Zheng Y, et al. ZBTB16 overexpression enhances white adipogenesis and induces brown-like adipocyte formation of bovine white intramuscular preadipocytes. Cell Physiol Biochem. 2018;48(6):2528–2538. 30121655 10.1159/000492697

[72] Meissburger B, Ukropec J, Roeder E, et al. Adipogenesis and insulin sensitivity in obesity are regulated by retinoid-related orphan receptor gamma. EMBO Mol Med. 2011;3(11):637–651. . 21853531 10.1002/emmm.201100172 PMC3377107

[73] Walton RG, Zhu B, Unal R, et al. Increasing adipocyte lipoprotein lipase improves glucose metabolism in high fat diet-induced obesity. J Biol Chem. 2015;290(18):11547–11556. 25784555 10.1074/jbc.M114.628487 PMC4416858

[74] Helfer G, Wu QF. Chemerin: a multifaceted adipokine involved in metabolic disorders. J Endocrinol. 2018;238(2):R79–R94. 29848608 10.1530/JOE-18-0174 PMC6026924

[75] Wu Y, Smas CM. Wdnm1-like, a new adipokine with a role in MMP-2 activation. Am J Physiol Endocrinol Metab. 2008;295(1):E205–15. 18492766 10.1152/ajpendo.90316.2008 PMC2493586

[76] Jiang Y, Berry DC, Graff JM. Distinct cellular and molecular mechanisms for beta3 adrenergic receptor-induced beige adipocyte formation. Elife. 2017;6. doi:10.7554/eLife.30329 PMC5667933 29019320

[77] Zhang CM, Chi X, Wang B, et al. Downregulation of STEAP4, a highly-expressed TNF-alpha-inducible gene in adipose tissue, is associated with obesity in humans. Acta Pharmacol Sin. 2008;29(5):587–592. . 18430367 10.1111/j.1745-7254.2008.00793.x

[78] Tomaru T, Steger DJ, Lefterova MI, et al. Adipocyte-specific expression of murine resistin is mediated by synergism between peroxisome proliferator-activated receptor gamma and CCAAT/enhancer-binding proteins. J Biol Chem. 2009;284(10):6116–6125. 19126543 10.1074/jbc.M808407200 PMC2649096

[79] Nishizuka M, Honda K, Tsuchiya T, et al. RGS2 promotes adipocyte differentiation in the presence of ligand for peroxisome proliferator-activated receptor gamma. J Biol Chem. 2001;276(32):29625–29627. 11418611 10.1074/jbc.C100272200

[80] Jiao H, Kulyte A, Naslund E, et al. Whole-exome sequencing suggests LAMB3 as a susceptibility gene for morbid obesity. Diabetes. 2016;65(10):2980–2989. . 27431458 10.2337/db16-0522

[81] Yamashita-Sugahara Y, Tokuzawa Y, Nakachi Y, et al. Fam57b (family with sequence similarity 57, member B), a novel peroxisome proliferator-activated receptor gamma target gene that regulates adipogenesis through ceramide synthesis. J Biol Chem. 2013;288(7):4522–4537. . 23275342 10.1074/jbc.M112.440792 PMC3576059

[82] Ralston JC, Mutch DM. SCD1 inhibition during 3T3-L1 adipocyte differentiation remodels triacylglycerol, diacylglycerol and phospholipid fatty acid composition. Prostaglandins Leukot Essent Fatty Acids. 2015;98:29–37. 25959085 10.1016/j.plefa.2015.04.008

[83] Shearin AL, Monks BR, Seale P, et al. Lack of AKT in adipocytes causes severe lipodystrophy. Mol Metab. 2016;5(7):472–479. 27408773 10.1016/j.molmet.2016.05.006 PMC4921941

[84] Monteiro MC, Wdziekonski B, Villageois P, et al. Commitment of mouse embryonic stem cells to the adipocyte lineage requires retinoic acid receptor beta and active GSK3. Stem Cells Dev. 2009;18(3):457–463. . 18690793 10.1089/scd.2008.0154

[85] Markussen LK, Winther S, Wicksteed B, et al. GSK3 is a negative regulator of the thermogenic program in brown adipocytes. Sci Rep. 2018;8(1):3469. 29472592 10.1038/s41598-018-21795-y PMC5823915

[86] Marquez MP, Alencastro F, Madrigal A, et al. The role of cellular proliferation in adipogenic differentiation of human adipose tissue-derived mesenchymal stem cells. Stem Cells Dev. 2017;26(21):1578–1595. . 28874101 10.1089/scd.2017.0071 PMC5662072

[87] Otto TC, Lane MD. Adipose development: from stem cell to adipocyte. Crit Rev Biochem Mol Biol. 2005;40(4):229–242. 16126487 10.1080/10409230591008189

[88] Linhart HG, Ishimura-Oka K, DeMayo F, et al. C/EBPalpha is required for differentiation of white, but not brown, adipose tissue. Proc Natl Acad Sci U S A. 2001;98(22):12532–12537. . 11606718 10.1073/pnas.211416898 PMC60088

[89] Bauer RC, Yenilmez BO, Rader DJ. Tribbles-1: a novel regulator of hepatic lipid metabolism in humans. Biochem Soc Trans. 2015;43(5):1079–1084. 26517927 10.1042/BST20150101 PMC4613491

[90] Chamorro-Garcia R, Shoucri BM, Willner S, et al. Effects of perinatal exposure to dibutyltin chloride on fat and glucose metabolism in mice, and molecular mechanisms, in vitro. Environ Health Perspect. 2018;126(5):057006. 29787037 10.1289/EHP3030 PMC6072003

[91] Franck N, Gummesson A, Jernas M, et al. Identification of adipocyte genes regulated by caloric intake. J Clin Endocrinol Metab. 2011;96(2):E413–8. . 21047925 10.1210/jc.2009-2534

[92] Martinez B, Khudyakov J, Rutherford K, et al. Adipose transcriptome analysis provides novel insights into molecular regulation of prolonged fasting in northern elephant seal pups. Physiol Genomics. 2018;50(7):495–503. 29625017 10.1152/physiolgenomics.00002.2018 PMC6087879

[93] Heinonen I, Kalliokoski KK, Hannukainen JC, et al. Organ-specific physiological responses to acute physical exercise and long-term training in humans. Physiology (Bethesda). 2014;29(6):421–436. 25362636 10.1152/physiol.00067.2013

[94] Tandon P, Wafer R, Minchin JEN. Adipose morphology and metabolic disease. J Exp Biol. 2018;221(PtSuppl 1). doi:10.1242/jeb.164970 29514883

[95] Berry DC, Stenesen D, Zeve D, et al. The developmental origins of adipose tissue. Development. 2013;140(19):3939–3949. 24046315 10.1242/dev.080549 PMC3775412

[96] Hepler C, Shan B, Zhang Q, et al. Identification of functionally distinct fibro-inflammatory and adipogenic stromal subpopulations in visceral adipose tissue of adult mice. Elife. 2018;7:e39636. doi: 10.7554/eLife.39636. PMC6167054 30265241

[97] Kim CY, Kim KH. Dexamethasone-induced selenoprotein S degradation is required for adipogenesis. J Lipid Res. 2013;54(8):2069–2082. 23687306 10.1194/jlr.M034603 PMC3708358

[98] Kulyte A, Ehrlund A, Arner P, et al. Global transcriptome profiling identifies KLF15 and SLC25A10 as modifiers of adipocytes insulin sensitivity in obese women. PLoS One. 2017;12(6):e0178485. 28570579 10.1371/journal.pone.0178485 PMC5453532

[99] Cho YL, Min JK, Roh KM, et al. Phosphoprotein phosphatase 1CB (PPP1CB), a novel adipogenic activator, promotes 3T3-L1 adipogenesis. Biochem Biophys Res Commun. 2015;467(2):211–217. . 26449462 10.1016/j.bbrc.2015.10.004

[100] Capasso S, Alessio N, Di Bernardo G, et al. Silencing of RB1 and RB2/P130 during adipogenesis of bone marrow stromal cells results in dysregulated differentiation. Cell Cycle. 2014;13(3):482–490. . 24281253 10.4161/cc.27275 PMC3956544

[101] Choi YJ, Lee HW, Lee YS, et al. RRP12 is a crucial nucleolar protein that regulates p53 activity in osteosarcoma cells. Tumour Biol. 2016;37(4):4351–4358. 26499779 10.1007/s13277-015-4062-2

[102] Hansson B, Moren B, Fryklund C, et al. Adipose cell size changes are associated with a drastic actin remodeling. Sci Rep. 2019;9(1):12941. 31506540 10.1038/s41598-019-49418-0 PMC6736966

[103] Schmidt D, Schwalie PC, Ross-Innes CS, et al. A CTCF-independent role for cohesin in tissue-specific transcription. Genome Res. 2010;20(5):578–588. . 20219941 10.1101/gr.100479.109 PMC2860160

[104] Du C, Ma X, Meruvu S, et al. The adipogenic transcriptional cofactor ZNF638 interacts with splicing regulators and influences alternative splicing. J Lipid Res. 2014;55(9):1886–1896. 25024404 10.1194/jlr.M047555 PMC4617354

[105] Townson SM, Dobrzycka KM, Lee AV, et al. SAFB2, a new scaffold attachment factor homolog and estrogen receptor corepressor. J Biol Chem. 2003;278(22):20059–20068. . 12660241 10.1074/jbc.M212988200

[106] Marcon BH, Shigunov P, Spangenberg L, et al. Cell cycle genes are downregulated after adipogenic triggering in human adipose tissue-derived stem cells by regulation of mRNA abundance. Sci Rep. 2019;9(1):5611. . 30948750 10.1038/s41598-019-42005-3 PMC6449374

[107] Reichert M, Eick D. Analysis of cell cycle arrest in adipocyte differentiation. Oncogene. 1999;18(2):459–466. 9927202 10.1038/sj.onc.1202308

[108] Marcon BH, Holetz FB, Eastman G, et al. Downregulation of the protein synthesis machinery is a major regulatory event during early adipogenic differentiation of human adipose-derived stromal cells. Stem Cell Res. 2017;25:191–201. 29156375 10.1016/j.scr.2017.10.027

[109] Fu M, Rao M, Bouras T, et al. Cyclin D1 inhibits peroxisome proliferator-activated receptor γ-mediated adipogenesis through histone deacetylase recruitment. J Biol Chem. 2005;280(17):16934–16941. . 15713663 10.1074/jbc.M500403200

[110] Tang QQ, Otto TC, Lane MD. Mitotic clonal expansion: a synchronous process required for adipogenesis. Proc Natl Acad Sci U S A. 2003;100(1):44–49. 12502791 10.1073/pnas.0137044100 PMC140878

[111] Zuo Y, Qiang L, Farmer SR. Activation of CCAAT/enhancer-binding protein (C/EBP) alpha expression by C/EBP beta during adipogenesis requires a peroxisome proliferator-activated receptor-gamma-associated repression of HDAC1 at the C/ebp alpha gene promoter. J Biol Chem. 2006;281(12):7960–7967. 16431920 10.1074/jbc.M510682200

[112] Kuzmochka C, Abdou HS, Hache RJ, et al. Inactivation of histone deacetylase 1 (HDAC1) but not HDAC2 is required for the glucocorticoid-dependent CCAAT/enhancer-binding protein alpha (C/EBPalpha) expression and preadipocyte differentiation. Endocrinology. 2014;155(12):4762–4773. 25203139 10.1210/en.2014-1565

[113] Jin J, Iakova P, Jiang Y, et al. Transcriptional and translational regulation of C/EBPbeta-HDAC1 protein complexes controls different levels of p53, SIRT1, and PGC1alpha proteins at the early and late stages of liver cancer. J Biol Chem. 2013;288(20):14451–14462. . 23564453 10.1074/jbc.M113.460840 PMC3656300

